# University of Louisville

**Published:** 1851-08

**Authors:** 


### UNIVERSITY OF LOUISVILLE.

We invite the attention of Students of Medicine to the fact, that a course of lectures in the Medical Department of the University of Louisville will be commenced on the first October, and that the anatomical rooms will be open at the same time. This is a virtual extension of the lecture-term, by which, we know, many students are disposed to profit.

